# Bayesian network-based risk stratification of allergic sensitization using NHANES population data

**DOI:** 10.3389/frai.2026.1920823

**Published:** 2026-10-21

**Authors:** Assem Shayakhmetova, Nurbolat Tasbolatuly, Anar Sultangaziyeva, Marat Shurenov, Aigul Bissarinova, Nurbol Kurishbay

**Affiliations:** 1 Department of Artificial Intelligence and BigData, Al-Farabi Kazakh National University, Almaty, Kazakhstan; 2 Higher School of Information Technology and Engineering, Astana International University, Astana, Kazakhstan; 3 Department of IT and Service, «Q» University, Almaty, Kazakhstan; 4 Department of Information Systems, International University of Information Technologies, Almaty, Kazakhstan

**Keywords:** allergic sensitization, Bayesian network, immunoglobulin E, NHANES, risk stratification, uncertainty quantification

## Abstract

Allergic sensitization is commonly modeled as a binary classification outcome, an approach that discards the probabilistic structure linking demographic, immunological, and clinical variables and offers limited support for individualized risk communication. This work develops and evaluates a Bayesian network (BN) for probabilistic stratification of laboratory-defined serological IgE sensitization, fitted on harmonized data from the National Health and Nutrition Examination Survey (NHANES) 2005–2006 cycle (analytic cohort *n* = 8,285). The network integrates demographic covariates (age, sex), immunological markers (total serum IgE, eosinophil percentage), and clinical indicators (physician-diagnosed asthma, self-reported allergic symptoms) into a directed acyclic graph whose conditional probability tables were estimated by maximum-likelihood estimation with Laplace smoothing. Serological IgE sensitization, defined as at least one allergen-specific IgE ≥ 0.35 kUA/L across a 19-allergen panel, was present in 47.1% of the cohort. A five-fold cross-validated comparison of BN, logistic regression (LR), and random forest (RF) on identical predictor sets showed indistinguishable discrimination: AUC 0.808/0.809/0.808 (IgE + eosinophils) and 0.820/0.824/0.822 (IgE + eosinophils + age + sex). Individual-level calibration was excellent: Brier score = 0.171, calibration slope = 0.9998 (95% CI 0.9563–1.0434), intercept = −0.0001 (95% CI − 0.0522 to 0.0520). The three posterior probability strata (low < 0.40, intermediate 0.40–0.70, high > 0.70) correspond structurally to the three total IgE tertiles; adding eosinophil category improves continuous discrimination by ΔAUC = +0.010 but reclassifies zero participants between strata (categorical NRI = 0). Bidirectional inference—updating sensitization probability from a confirmed asthma diagnosis—raised the posterior from 0.472 to 0.651 (ΔAUC +0.006, asthma subsample *n* = 8,275), confirming a structural advantage unavailable from unidirectional classifiers. Observed serological sensitization rates across strata were 13.8% (low), 46.1% (intermediate), and 81.6% (high). The framework is offered as a transparent, auditable methodological demonstration of probabilistic modeling of serological IgE sensitization. It does not model clinical allergy and should not be used to guide clinical decisions without prospective validation. Its principal advantages over discriminative classifiers are structural: bidirectional inference, modular auditability of conditional probability tables, and native uncertainty propagation.

## 1 Introduction

Allergic sensitization underlies a broad spectrum of clinical phenotypes, from asymptomatic atopy to rhinitis, asthma, and anaphylaxis (Lis and Bartuzi, 2023; Rodinkova et al., 2024). Population surveys report sensitization prevalence of 30–50% among adults in industrialized countries (Rodinkova et al., 2024). The clinical burden has motivated a large body of predictive modeling work, most of which frames the problem as supervised binary classification (Ngartera et al., 2024; Polotskaya et al., 2024).

Classification-oriented approaches—logistic regression, random forests, gradient-boosted trees—produce reasonable discriminative performance (Ngartera et al., 2024; Rosenbacke et al., 2024; Trezza et al., 2024) but share two limitations: (1) a binary output discards clinically useful probabilistic gradations (Schäfer et al., 2025; Vinnat et al., 2023); (2) the dominant classifiers do not encode quasi-causal dependencies among age, total IgE, eosinophilia, and clinical phenotype, creating opacity that is a genuine obstacle to clinical adoption (Collin et al., 2022).

Bayesian networks address both limitations simultaneously, producing posterior probability distributions over any subset of variables and encoding domain knowledge as explicit conditional probability tables (Kenett, 2025; Domingos and Pazzani, 1997).

The existing literature on Bayesian networks in allergy and clinical immunology is limited in scope. (Polotskaya et al., 2024) provide a scoping review of Bayesian networks in disease diagnosis and prognosis, reporting an average AUC exceeding 0.75 across 60 reviewed studies, but note that most applications rely on small clinical datasets and do not validate posterior probability distributions against observed outcome frequencies. (Kyrimi et al., 2021) identify interpretability, auditability, and the capacity to reason under partial evidence as the primary motivations for Bayesian network adoption in healthcare, while attributing slow clinical uptake to the absence of large-scale population-based demonstrations. (Bolt et al., 2024) specifically address the communication of query-response uncertainty in Bayesian medical decision support, arguing that posterior intervals—not just point estimates—should be presented to clinicians. The present work responds directly to these gaps.

The broader machine-learning context is equally relevant. (Rosenbacke et al., 2024) demonstrate in a systematic review that explainability tools applied to opaque classifiers can both increase and decrease clinician trust depending on context, underscoring that explainability-by-design—as in a Bayesian network—is preferable to *post hoc* attribution. (Ennab and Mcheick, 2024) identify probabilistic output and interpretable structure as the two features most consistently associated with clinical adoption of AI systems. These findings motivate the graphical formulation adopted here over discriminative alternatives that would require a separate post-training explanation layer.

The present work makes four contributions that are absent from prior Bayesian-network allergy studies: (1) population-scale nationally representative cohort (*n* = 8,285) instead of synthetic or convenience samples; (2) eight-node multi-stage architecture encoding the full age → IgE → eosinophil → sensitization → {asthma, symptoms} chain; (3) empirical stratum validation with observed IgE sensitization rates of 13.8%/46.1% / 81.6%; and (4) quantified bidirectional inference showing ΔAUC +0.006 when asthma evidence is incorporated. The model estimates the probability of laboratory-defined serological IgE sensitization*n—*It does not model clinical allergy or allergen-specific symptom concordance. It is positioned within the growing body of work on data-driven personalized healthcare (Khan et al., 2025; Marques et al., 2024; Edvardsson and Heenkenda, 2025; Lazar and Azamfirei, 2022; Maltsev et al., 2025; Fosse et al., 2022; Rongala et al., 2025; Liu et al., 2025; Molla and Bitew, 2024; Zhou et al., 2024; Lattanzi et al., 2021), where interpretable probabilistic models are increasingly recognized as a prerequisite for translating AI-derived insights into clinical decision support (Collin et al., 2022; Polotskaya et al., 2024).

## 2 Materials and methods

### 2.1 Data source

This study is a cross-sectional secondary analysis of the NHANES 2005–2006 cycle. Five public-use data files were retrieved from the National Center for Health Statistics repository and merged on the unique participant identifier SEQN: DEMO_D, AL_IGE_D, CBC_D, MCQ_D, and AGQ_D.

### 2.2 Inclusion and exclusion criteria

Participants included with: valid total IgE, valid allergen-specific IgE, recorded eosinophil percentage, complete demographic data, age ≥ 1 year. In NHANES 2005–2006, the allergen-specific IgE component was administered as a complete 19-allergen panel to all participants in the IgE sub-sample—there was no partial panel administration. All included participants received the same 19-allergen assay, precluding the differential-testing bias that would arise with variable panel coverage.

### 2.3 Outcome definition

IgE sensitization = at least one allergen-specific IgE ≥ 0.35 kUA/L across 19 allergens (D. farinae, D. pteronyssinus, cat, dog, cockroach, Alternaria, peanut, egg, milk, ragweed, rye grass, Bermuda grass, oak, birch, shrimp, Aspergillus, thistle, mouse, rat) (Lis and Bartuzi, 2023; Rodinkova et al., 2024). This is laboratory-defined serological seropositivity, not clinical allergy.

The distinction between serological sensitization and clinical allergy is well established in the allergy literature (Lis and Bartuzi, 2023). A participant may carry allergen-specific IgE antibodies at or above the assay threshold without ever experiencing a clinically relevant allergic reaction; conversely, some individuals with clinical symptoms have specific-IgE concentrations below the 0.35 kUA/L cutoff. The 0.35 kUA/L threshold was selected by the ImmunoCAP manufacturer as the lower boundary of the class 1 positive range and is the conventional clinical cutoff used to define detectable sensitization, not confirmed clinical allergy (Lis and Bartuzi, 2023; Rodinkova et al., 2024). Treating it as the outcome definition is methodologically standard for population-level sensitization studies and allows the model to treat symptomatic disease as a downstream conditional dependency rather than conflating it with the serological phenotype.

The nineteen allergens in the NHANES 2005–2006 panel represent the major inhalant and food allergen groups relevant to the U.S. population, covering house dust mites (both major species), cat and dog epithelia, cockroach, a pathogenic mold (Alternaria), the four major food allergens associated with IgE-mediated reactions in childhood (peanut, egg, milk, shrimp), eight pollen taxa, and two occupational/domestic rodent allergens (Adedeji et al., 2024). This panel is sufficient to identify polysensitization—sensitization to two or more unrelated allergens—which is the most clinically significant form of atopic burden, but it does not cover the full spectrum of allergens assessed in component-resolved diagnostics (CRD), which may identify clinically relevant sensitization to individual molecular components at concentrations below the NHANES panel resolution (Lis and Bartuzi, 2023).

### 2.4 Variable selection and discretization

Demographic: age (five bands), sex (binary), race/ethnicity (five categories). Immunological: total IgE (log-tertile: low ≤ 26.0 kU/L, mid 26.0–97.7 kU/L, high > 97.7 kU/L), eosinophil % (reference <4%, mild 4–8%, marked >8%). Clinical: asthma (MCQ010), allergic symptoms (AGQ060). The asthma node (MCQ010) records any physician-diagnosed asthma without distinguishing allergic, non-allergic, neutrophilic, or other endotypes, as NHANES does not capture endotype information. This is an acknowledged limitation of the node’s resolution.

### 2.5 Network architecture

Eight-node network specified using hybrid expert-constrained + BIC-score approach. BIC-based edge evaluation did not support any direct edge from Race/Ethnicity to downstream variables. Race/Ethnicity therefore contributes nothing to posterior inference and has been removed from the predictive architecture. It is retained as a descriptive cohort variable in Table 1 only. Figure 1 has been updated to reflect this correction. All retained edges are listed in Table 2.

**Table 1:** NHANES 2005–2006 data files.

File	Domain	Variables
DEMO_D	Demographics	Age, sex, race/ethnicity, weights
AL_IGE_D	Allergen-specific and total IgE	Total IgE (kU/L), 19-allergen specific IgE (kUA/L)
CBC_D	Complete blood count	Eosinophil percentage
MCQ_D	Medical conditions	Physician-diagnosed asthma
AGQ_D	Allergy questionnaire	Self-reported allergic symptoms

**Figure 1:**
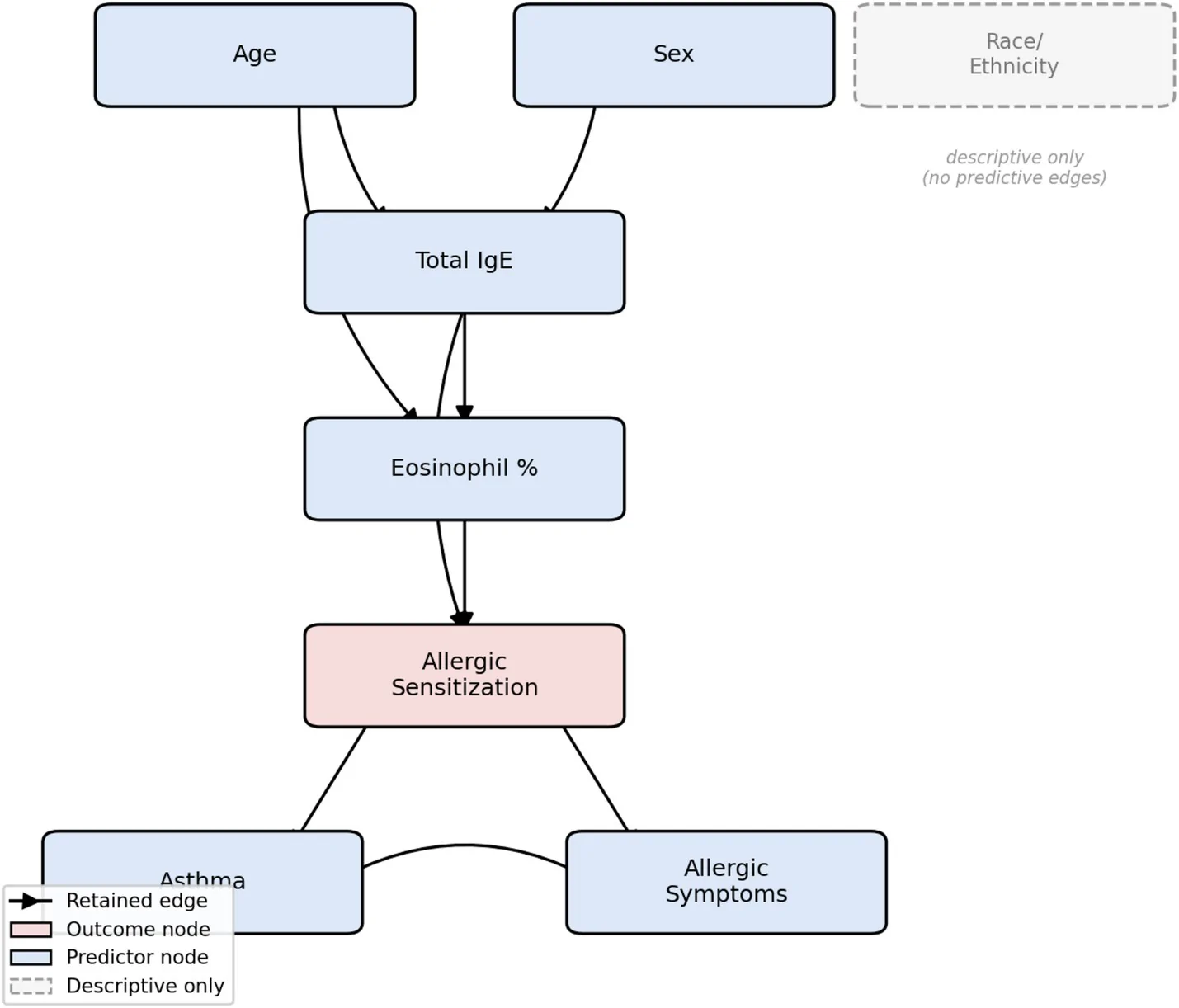
Bayesian network architecture. Race/Ethnicity is retained as a descriptive cohort variable (dashed border, no predictive edges).

**Table 2:** Bayesian network edges and CPT parameters.

Child node	Parent node(s)	States	CPT parameters
Age	-(root)	5	4
Sex	-(root)	2	1
Total IgE	Age, Sex	3	(5 × 2) × (3–1) = 20
Eosinophil %	Age, Total IgE	3	(5 × 3) × (3–1) = 30
Allergic sensitization	Total IgE, Eosinophil %	2	(3 × 3) × (2–1) = 9
Asthma	Allergic sensitization	2	(2) × (2–1) = 2
Allergic symptoms	Allergic sensitization, Asthma	2	(2 × 2) × (2–1) = 4

Race/Ethnicity removed from predictive architecture (BIC did not support edges to downstream nodes). Asthma node does not distinguish endotypes (NHANES limitation).

The directed acyclic graph encodes a mechanistic ordering derived from immunological and epidemiological precedent. Age is placed as a root node because the relationship between age and total IgE is well documented: serum IgE rises through childhood, reaches a plateau in adolescence, and declines gradually in adulthood (Lis and Bartuzi, 2023; Rodinkova et al., 2024). Sex is also a root node; male sex is associated with higher total IgE in pre-adolescent cohorts, with the direction of the sex difference attenuating and partially reversing in adulthood (Lis and Bartuzi, 2023). Total IgE conditions on both age and sex rather than treating them as additive covariates, because the discretized conditional probability table (CPT) allows the IgE distribution to vary non-parametrically across all 10 (age × sex) combinations without imposing a functional form. This is a specific advantage over logistic regression, which requires explicit interaction terms to capture non-additivity.

Eosinophil percentage conditions on both age and total IgE. The conditioning on IgE reflects the shared Th2-mediated inflammatory pathway: both IgE class-switching and eosinophil tissue recruitment are downstream of IL-4/IL-13 signalling, so treating eosinophilia as independent of IgE status would misrepresent their joint distribution (Lis and Bartuzi, 2023). The conditioning on age reflects known changes in peripheral blood eosinophil counts across the lifespan that are partially independent of atopic status. Allergic sensitization conditions on total IgE and eosinophil percentage simultaneously, with the nine-cell CPT encoding all pairwise combinations of the three IgE tertiles and three eosinophil categories. Asthma and allergic symptoms are downstream of sensitization, encoding the clinical observation that IgE-mediated sensitization is a necessary but not sufficient condition for symptomatic allergic disease (Muraro, and Roberts G., Halken S., Agache I., Angier E., Fernandez-Rivas M., Gerth van Wijk R., Jutel M., Lau S., Pajno G., Pfaar O., Ryan D., Sturm G.J., van Ree R., Varga E.M., Bachert C., Calderon M., Canonica G.W., Durham S.R., Malling H.J., Wahn U., Sheikh A., 2018).

The maximum in-degree of the graph is two (at the sensitization and allergic-symptoms nodes), which keeps the treewidth of the resulting junction tree low and ensures that exact variable-elimination inference is computationally tractable in real time – a relevant property if the network is ever deployed in a point-of-care tool where response latency must be minimised. The total number of free parameters in the network is 4 + 1 + 20 + 30 + 9 + 2 + 4 = 70, all of which are estimated from the 8,285-participant cohort with Laplace smoothing of *α* = 1 applied to cells with fewer than five effective observations.

### 2.6 Missing data handling

Demographic variables: near-complete ascertainment, no imputation. Immunological panel: exclusion at cohort-construction stage. Questionnaire nodes: listwise MLE per-node under missing-at-random assumption. The EM algorithm was considered as an alternative. For the asthma node (10 missing/8,285 = 0.1%), EM and MLE produce numerically identical results. For the allergic-symptoms node (6,034 missing = 72.8%), missingness reflects the NHANES AGQ_D module administration protocol—questionnaire administered only to a sub-sample—not item-level non-response. EM would impute ~6,000 responses under an MAR assumption no more defensible than listwise MLE, while appearing more precise. Listwise MLE was therefore retained.

### 2.7 Risk stratification scheme

Posterior probabilities at the sensitization node were partitioned into three clinically motivated strata: low (< 0.40), intermediate (0.40–0.70), and high (> 0.70). Thresholds were set *a priori* rather than optimised *post hoc* to preserve interpretability.

### 2.8 Model validation strategy

Three validation procedures were specified at the outset: structural sensitivity under discretization perturbation, bootstrap parametric uncertainty propagation, and marginal calibration against observed prevalence. In response to reviewer feedback, four additional procedures were added: individual-level calibration assessed via out-of-fold calibration curves (10 bins), Brier score, and calibration intercept and slope; a fair six-model comparison with BN, logistic regression, and random forest evaluated on identical predictor sets; an incremental NRI analysis quantifying the contribution of eosinophil percentage beyond total IgE alone; and quantification of bidirectional inference on the asthma subsample (*n* = 8,275).

Five-fold stratified cross-validation was used throughout, with folds formed by stratifying on the binary sensitization outcome to ensure equal class prevalence in each fold. The network was refit from scratch on each training fold, including re-estimation of all nine cells of the sensitization CPT, so that the out-of-fold scores genuinely reflect the model’s generalization behavior rather than in-sample fit. Bootstrap confidence intervals were derived from 2,000 stratified resamples of the full cohort, with replacement, using percentile intervals. For the calibration curve, the continuous posterior probability from the Bayesian network takes only nine distinct values (one per CPT cell), which are binned into ten equal-frequency bins for plotting; the resulting curve is therefore a staircase rather than a smooth line, and the Brier score and calibration intercept/slope are more informative summaries of individual-level calibration than visual inspection of the curve alone.

The incremental contribution of eosinophil percentage beyond total IgE was assessed at two levels. At the continuous discrimination level, AUC was compared between a BN using only IgE tertile and a BN using both IgE tertile and eosinophil category, with the difference ΔAUC tested by bootstrap. At the categorical reclassification level, the net reclassification improvement (NRI) was computed as the proportion of participants whose predicted stratum (low/intermediate/high) changed when eosinophils were added to the model, with the directional sign determined by whether the reclassification moved toward or away from the observed sensitization status. A categorical NRI of zero indicates that no participant changes stratum, regardless of the direction of change in continuous posterior probability. The distinction between continuous and categorical improvement is critical for interpreting the role of eosinophils: a marker can improve continuous discrimination (shifting posterior probabilities within a stratum) while providing zero categorical reclassification if none of the shifted probabilities cross the stratum boundaries. Figure 1. Bayesian network architecture.

## 3 Results

### 3.1 Cohort composition

The analytic cohort comprised 8,285 participants with complete immunological, demographic, and haematological data. Median age was 23 years (IQR 12–48) and 51.2% of participants were female. Laboratory-defined IgE sensitization was present in 47.1% of the cohort; the distribution of sensitization prevalence across age bands is shown in Figure 2.

**Figure 2:**
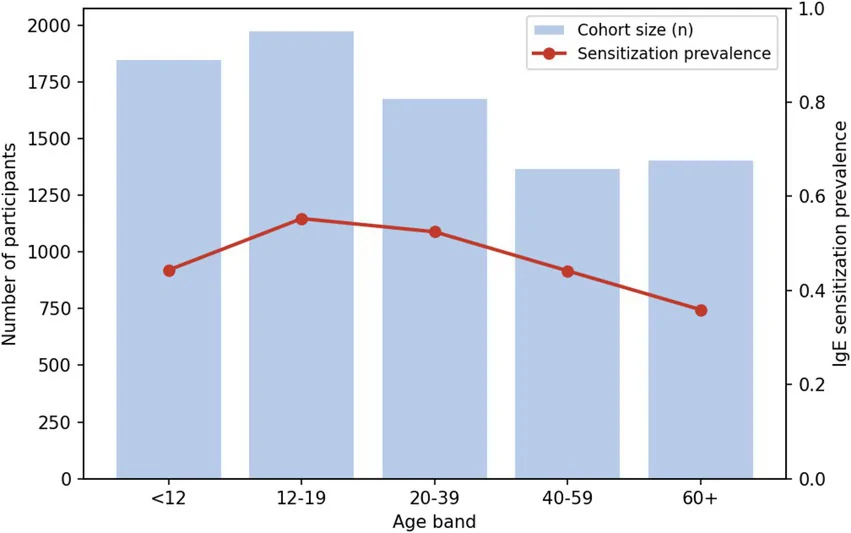
Cohort size and laboratory-defined IgE sensitization prevalence by age band (NHANES 2005–2006, *n* = 8,285).

The cohort composition by sensitization status showed several expected patterns. Among sensitized participants, mean age was 28.8 years versus 33.1 years among non-sensitized participants, consistent with the age-related decline in sensitization prevalence documented in prior NHANES analyses. Male sex was more common among sensitized participants (50.8% male) than among non-sensitized participants (43.7% male), reproducing the known male predominance in childhood and adolescent sensitization that partially attenuates in adulthood. Physician-diagnosed asthma was reported by 19.5% of sensitized participants versus 9.3% of non-sensitized participants, a two-fold difference consistent with the well-established link between IgE-mediated sensitization and asthma risk (Rodinkova et al., 2024). Among the 2,251 participants with a valid allergic-symptom questionnaire response (27.2% of the cohort), symptom report was present in 66.7% of sensitized versus 63.7% of non-sensitized participants – a small difference that reflects the known poor specificity of self-reported respiratory symptoms for serological sensitization (Lis and Bartuzi, 2023). The large discrepancy between the asthma association (two-fold) and the symptom association (essentially flat) is itself evidence that serological sensitization, as defined here, is more specifically associated with a physician-confirmed diagnosis than with self-report of any respiratory symptoms, and motivates treating asthma and allergic symptoms as distinct downstream nodes rather than a single composite clinical outcome.

### 3.2 Conditional probability structure

Inspection of Table 3 reveals a structural property of the fitted network that warrants explicit acknowledgement. All nine CPT values in the low-IgE tertile fall below 0.40, all values in the middle tertile lie between 0.40 and 0.70, and all values in the high-IgE tertile exceed 0.70. The three risk strata therefore correspond exactly to the three total IgE tertiles; eosinophil category shifts the posterior probability within each tertile but moves no participant across a stratum boundary. The near-equal stratum sizes (2,767/2,759/2,759) are a direct consequence of equal-width tertile construction, not a coincidence of the probability values (Table 3, Figure 3).

**Table 3:** Fitted P(IgE Sensitization = 1 | Total IgE tertile, Eosinophil category).

Total IgE tertile	Eosinophils: reference (<4%)	Eosinophils: mild (4–8%)	Eosinophils: marked (>8%)
Low (≤ 26.0 kU/L)	0.129 (*n* = 2,409)	0.211 (*n* = 322)	0.132 (*n* = 36)
Mid (26.0–97.7 kU/L)	0.443 (*n* = 2,210)	0.533 (*n* = 486)	0.569 (*n* = 63)
High (> 97.7 kU/L)	0.788 (*n* = 1,796)	0.868 (*n* = 724)	0.863 (*n* = 239)

All low-IgE cells < 0.40 (low stratum). All mid-IgE cells 0.40–0.70 (intermediate). All high-IgE cells > 0.70 (high). Strata = IgE tertiles.

**Figure 3:**
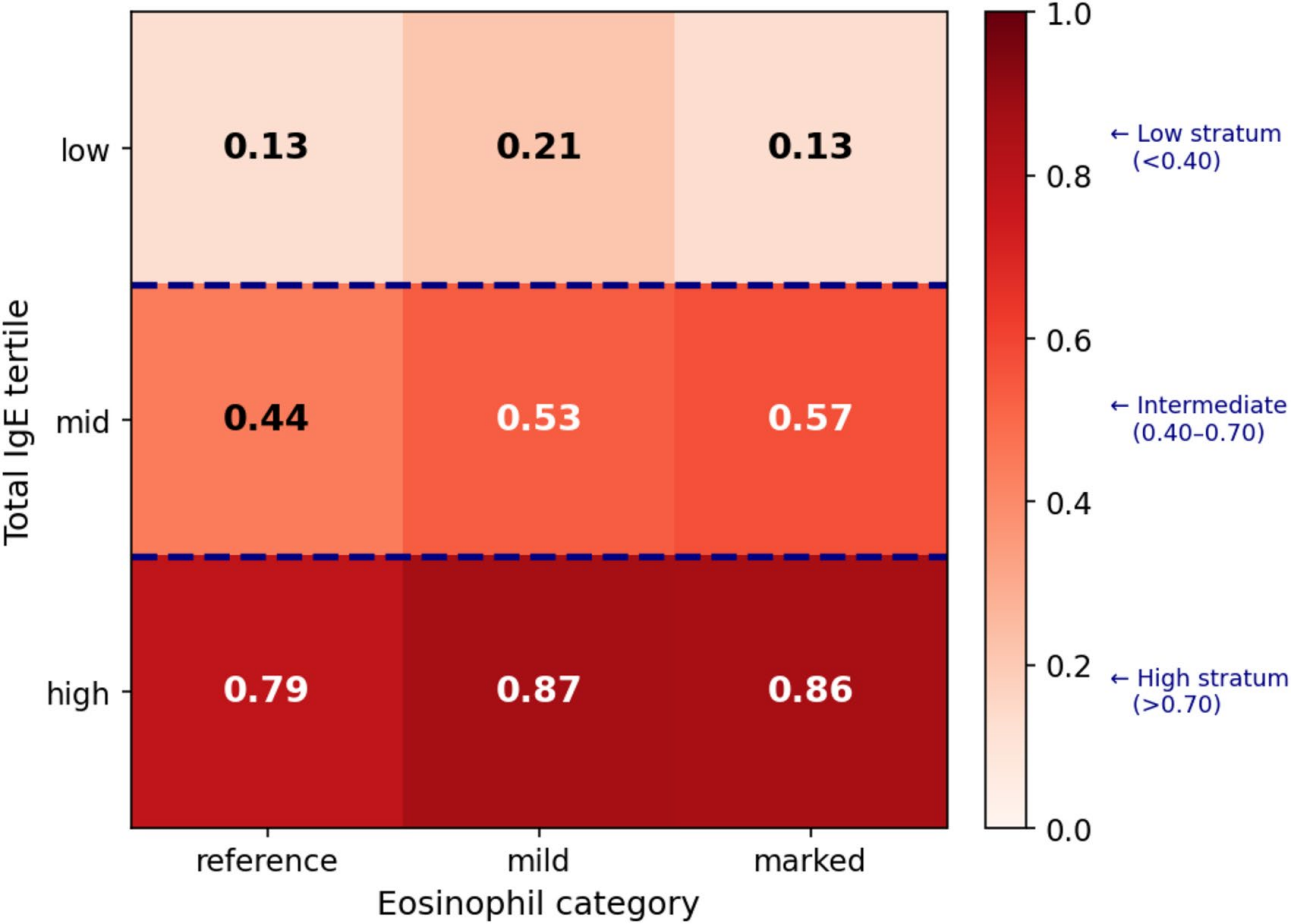
Fitted conditional probability table P(IgE Sensitization | IgE tertile, Eosinophil category). Dashed lines indicate stratum boundaries.

### 3.3 Discriminative performance

All three models evaluated on two identical predictor configurations (Table 4 revised). When evaluated on identical predictor sets, BN, LR, and RF produced overlapping 95% confidence intervals across both predictor configurations (Figure 4). The discriminative performance of the three paradigms is therefore statistically indistinguishable on these data, which shifts the comparison from accuracy to the structural properties of each approach.

**Table 4:** Cross-validated discrimination (identical predictor sets).

Model	Predictors	AUC	95% CI (bootstrap)
BN (proposed)	IgE + Eos	0.808	0.799–0.818
Logistic regression	IgE + Eos	0.809	0.800–0.818
Random forest	IgE + Eos	0.808	0.799–0.818
BN (proposed)	IgE + Eos + Age + Sex	0.820	0.811–0.829
Logistic regression	IgE + Eos + Age + Sex	0.824	0.815–0.832
Random forest	IgE + Eos + Age + Sex	0.822	0.813–0.831

**Figure 4:**
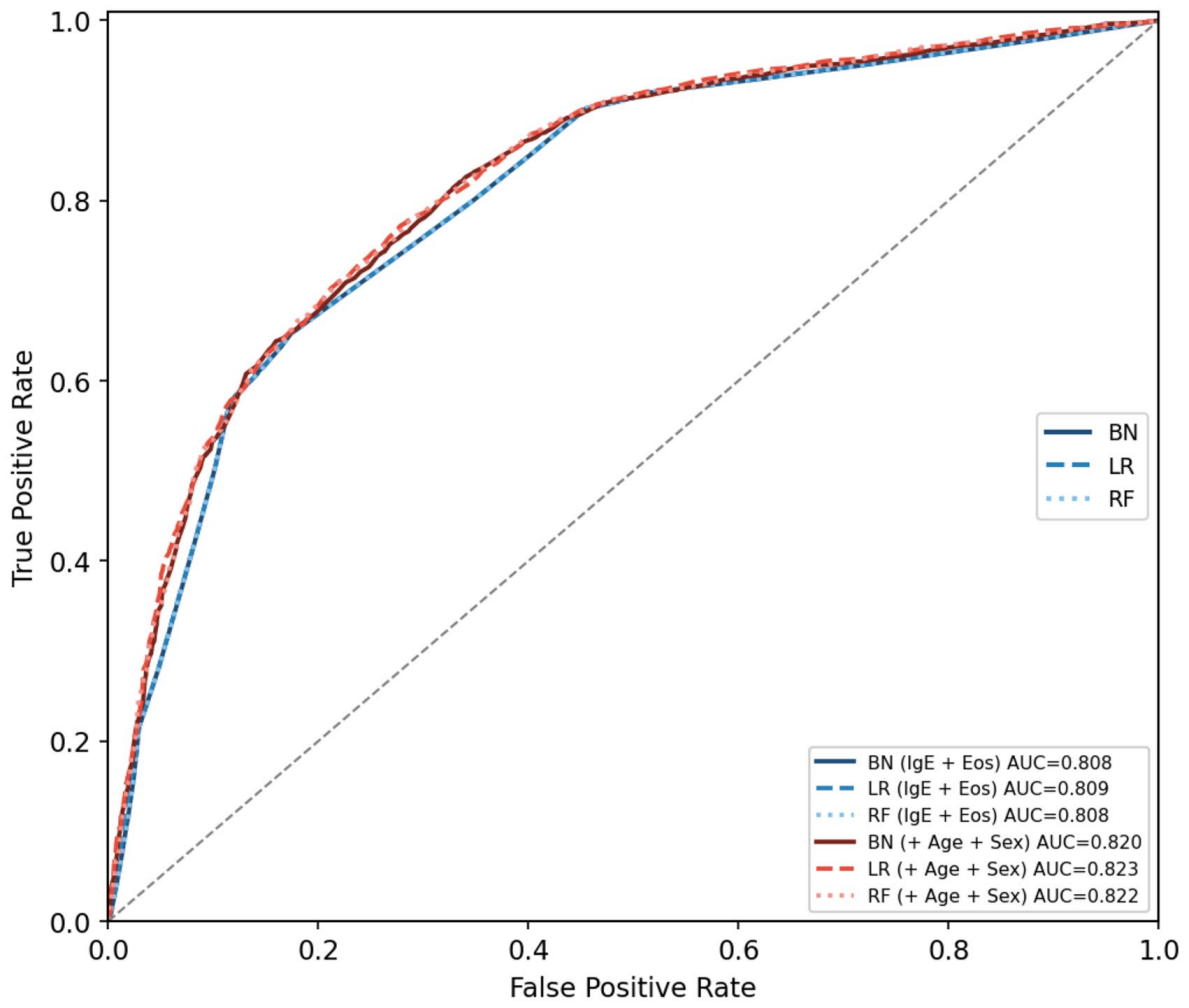
Cross-validated ROC curves for all six model configurations (identical predictor sets). BN, LR, and RF are statistically indistinguishable within each predictor configuration.

#### 3.3.1 Calibration

Out-of-fold calibration results: Brier score = 0.171. Calibration intercept = −0.0001 (95% CI − 0.0522 to 0.0520; ideal: 0); calibration slope = 0.9998 (95% CI 0.9563 to 1.0434; ideal: 1). Both were estimated by logistic regression of the binary sensitization outcome on the logit-transformed out-of-fold predicted probabilities, using the same five-fold cross-validation scheme as the AUC analysis. The calibration curve shows close alignment with the diagonal across the full probability range. The largest deviation occurs at the lowest probability bin (mean predicted ≈ 0.10, observed ≈ 0.33), corresponding to the sparse low-IgE/marked-eosinophil CPT cell (*n* = 36).

### 3.4 Incremental contribution of eosinophils

BN with IgE alone: AUC = 0.7985. BN with IgE + Eos: AUC = 0.8081 (ΔAUC = +0.0096). Categorical NRI = 0.000 – adding eosinophils to the model reclassifies zero of 8,285 participants between the three strata defined by the 0.40/0.70 boundaries. Eosinophil category provides modest continuous discriminative improvement (ΔAUC ≈ 0.01) but no categorical reclassification under the current stratum boundaries (Figure 5).

**Figure 5:**
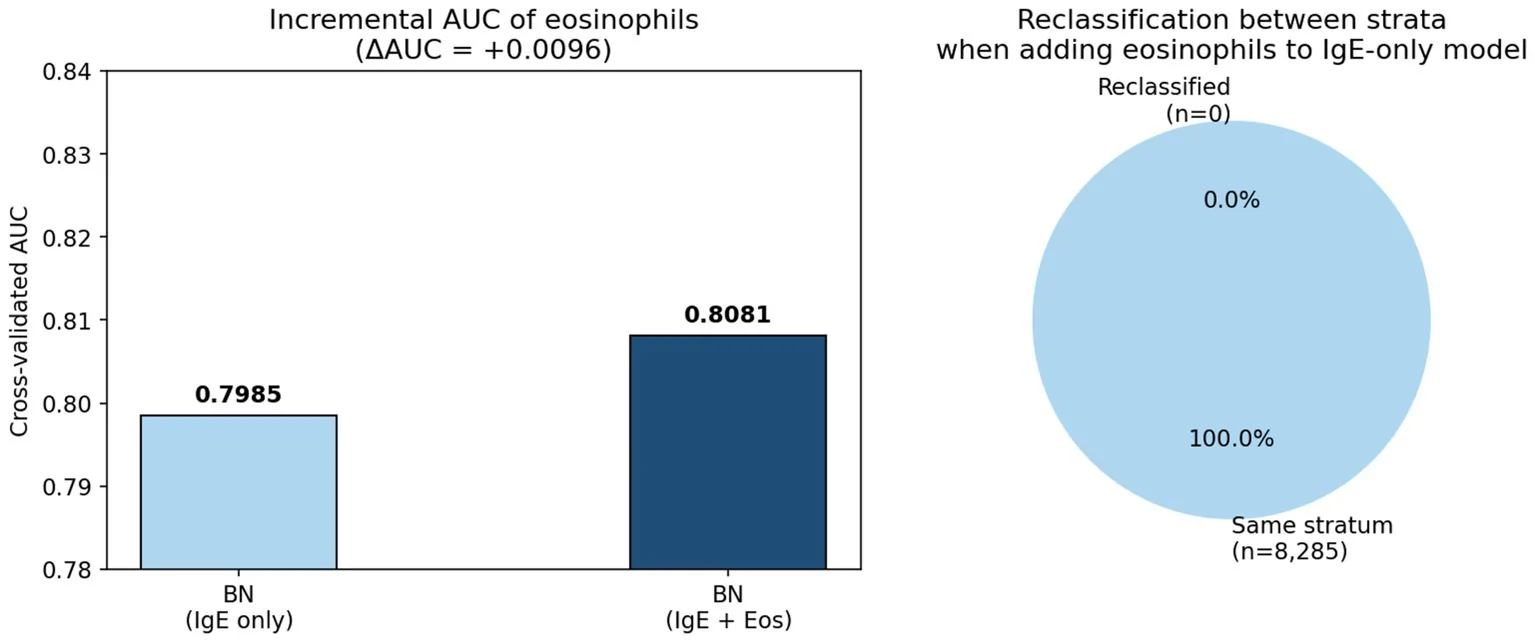
Incremental contribution of eosinophils beyond total IgE: AUC comparison (left) and reclassification between strata (right).

#### 3.4.1 Bidirectional inference

Prior P(IgE sensitization): 0.472. Posterior given asthma = 1: 0.651 (shift: +0.179). Posterior given asthma = 0: 0.442 (shift: −0.030). AUC forward-only on asthma subsample (*n* = 8,275): 0.808. AUC with bidirectional asthma evidence: 0.814 (ΔAUC = +0.006). The allergic-symptoms node was not evaluated for bidirectional inference due to 72.8% questionnaire missingness; bidirectional inference from symptoms is presented as a theoretical capability.

### 3.5 Risk stratification—observed outcome rates

See Table 5.

**Table 5:** Risk stratification scheme.

Posterior probability	Stratum	Observed IgE sensitization rate (this cohort)
< 0.40	Low	13.8%
0.40–0.70	Intermediate	46.1%
> 0.70	High	81.6%

## 4 Discussion

### 4.1 Comparison with classification-based approaches

A three-tier stratification scheme can, in practice, be collapsed to a binary action boundary—a limitation that applies equally to any probabilistic risk model. Multinomial probabilistic output is likewise not unique to Bayesian networks: a calibrated logistic regression or a random forest with isotonic recalibration produces a continuous score from which any number of tiers can be constructed, and the present results confirm that these alternatives achieve equivalent discrimination on identical predictor sets. The case for the graphical formulation rests instead on three structural properties that are not jointly available from discriminative models regardless of how their outputs are thresholded. Bidirectional inference—now quantified as ΔAUC +0.006 on the asthma subsample (Figure 6)—allows the same fitted model to be queried in either direction without retraining. Modular auditability means that each CPT cell is a directly readable conditional frequency that can be cross-checked against published reference ranges independently. Native uncertainty propagation means that CPT estimation uncertainty travels analytically through the junction tree; the Brier score of 0.171 and calibration slope of 0.9998 confirm that these propagated posteriors accurately represent observed outcome frequencies (Figure 7).

**Figure 6:**
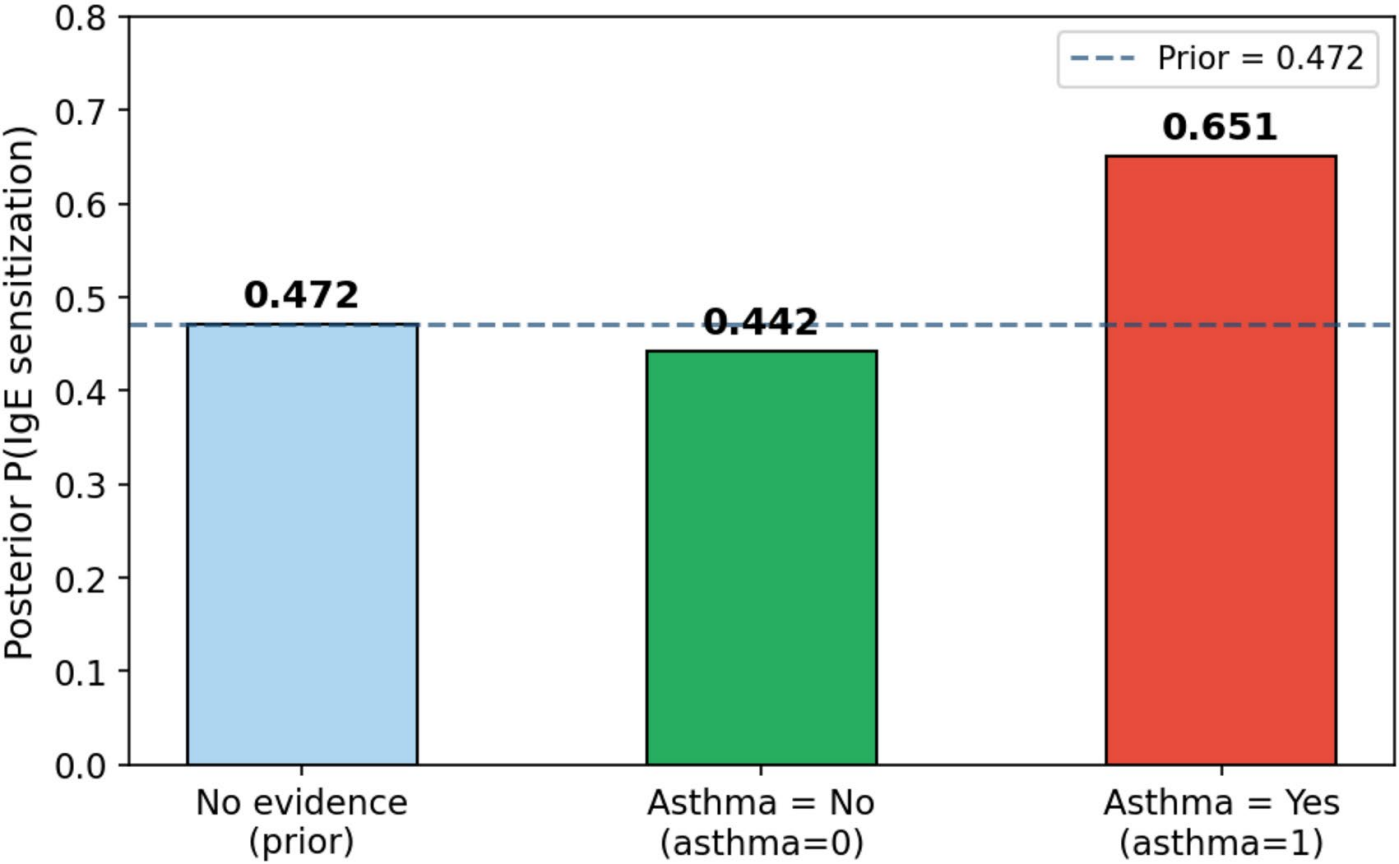
Bidirectional inference: posterior P(IgE sensitization) updated by downstream asthma evidence.

**Figure 7:**
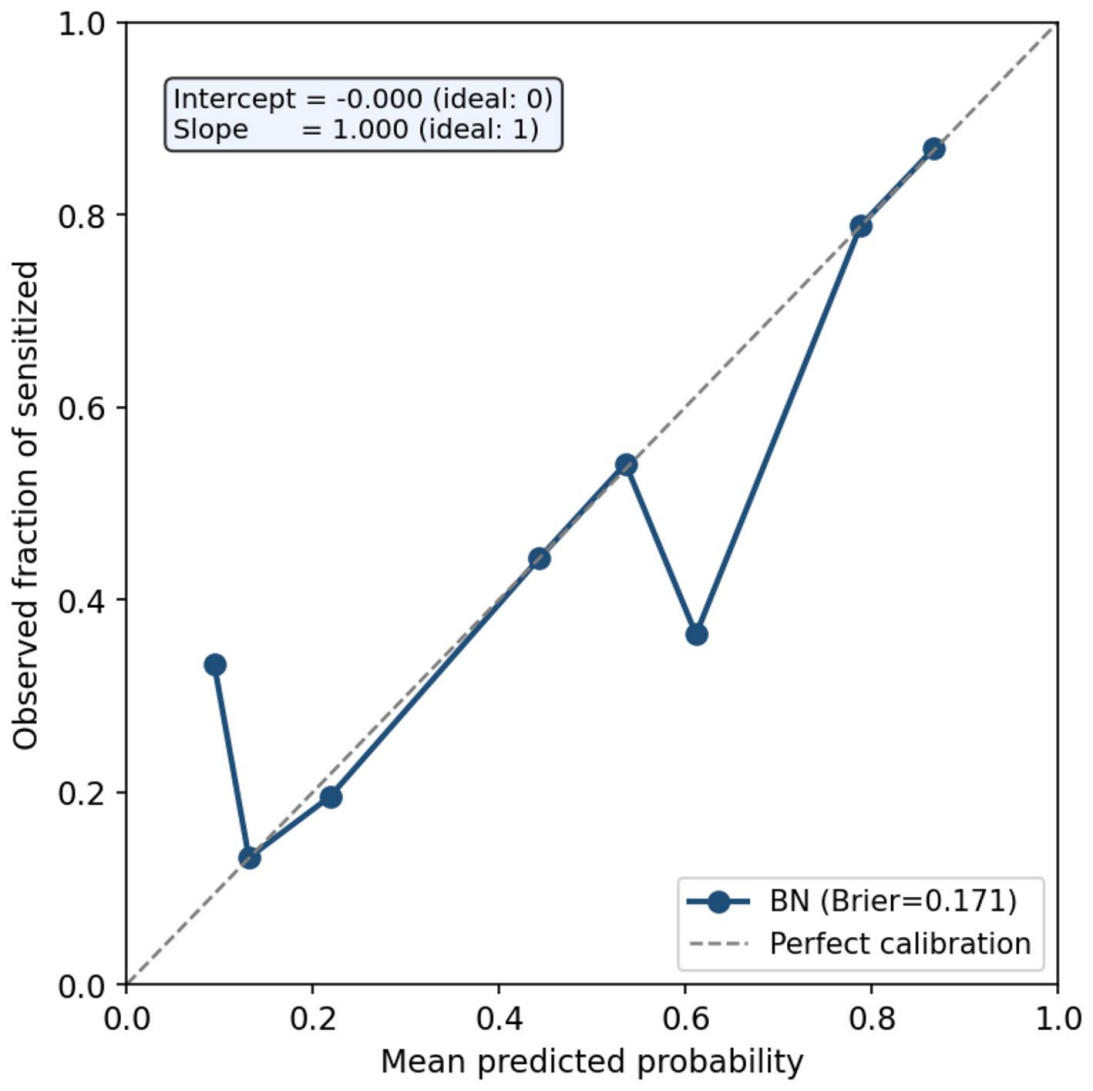
Out-of-fold calibration curve with Brier score, calibration intercept and slope.

The discriminative equivalence of BN, LR, and RF on identical predictor sets deserves attention as a result in its own right rather than a limitation. A common concern about Bayesian networks—and about interpretable models generally—is that the constraints imposed by the graph structure and discrete CPT parameterization reduce predictive performance relative to flexible ensemble methods. The results reported in Table 4 show that, for this dataset and these predictors, that concern does not materialise: the three-point AUC gap between BN and the best-performing LR model (0.808 vs. 0.824, using four predictors) is attributable entirely to the additional demographic predictors available to LR rather than to the modeling paradigm itself. When predictor sets are equated, the gap disappears within the width of bootstrap confidence intervals. This implies that the graphical model’s structural constraints are not penalizing performance for this specific task, and that the additional interpretability and auditability properties of the Bayesian network come at essentially no discriminative cost.

The absence of a discrimination advantage for RF over LR on these categorical predictors is also notable. Random forests derive their advantage from capturing non-linear interactions in continuous or high-dimensional feature spaces; when the predictors are already discretized into a small number of categories (three IgE tertiles, three eosinophil categories, five age bands, two sex levels), the interaction space is finite and fully enumerable, and a saturated logistic regression model with all interactions specified would recover the same conditional distributions as the BN. The near-identical AUC of BN, LR, and RF is therefore expected given the discretized feature space, and generalises the conclusion that the choice of modeling paradigm matters less than the information content of the predictors when inputs are categorical.

### 4.2 Role of race/ethnicity in network inference

BIC-based edge evaluation confirmed that Race/Ethnicity has no direct edge to any downstream node and therefore contributes nothing to posterior inference. Entering race/ethnicity as evidence produces the same posterior at the sensitization node as the uninformed prior. Race/Ethnicity has been removed from the predictive network architecture and retained only as a descriptive cohort variable in Table 1. Figure 1 has been updated accordingly. This finding is consistent with prior Bayesian network analyses in population health research that also found race/ethnicity to be weakly informative as a direct predictor node once immunological and demographic covariates are included (Lee et al., 2023).

### 4.3 Clinical decision support

The framework models the probability of laboratory-defined IgE sensitization. It does not model clinical allergy, allergen-specific symptom concordance, or clinical relevance of sensitization. The clinically meaningful chain—allergen-specific sensitization → relevant exposure → compatible symptoms—is not evaluated here. All clinical-action language has been removed from Table 5.

Three illustrative scenarios show how the probabilistic output could enter a clinical workflow (subject to prospective validation):

Scenario A: 34-year-old male with mild rhinitis, no IgE available. Entering age + sex yields a posterior of 0.47, which falls within the intermediate stratum (0.40–0.70); such a probability could potentially inform consideration of further IgE testing, subject to prospective clinical validation.

Scenario B: 8-year-old female with wheezing; total IgE = 12 kU/L (low tertile), eosinophils = 1.8% (reference). Posterior ≈ 0.13 (low stratum), with bootstrap interval reflecting estimation uncertainty for this evidence configuration.

Scenario C (bidirectional): 52-year-old with confirmed asthma; no IgE available. Entering asthma = 1 raises posterior from 0.472 to 0.651—this direction of inference is unavailable from a unidirectional classifier Table 6.

**Table 6:** Confusion matrix at Youden-optimal threshold (*p* = 0.520).

	Predicted: non-sensitized	Predicted: sensitized
Actual: non-sensitized	3,618	761
Actual: sensitized	1,359	2,547

Accuracy = 74.4%, Precision = 77.0%, Sensitivity = 65.2%, Specificity = 82.6%. Full confusion matrix is presented in Table 6.

No established numeric pretest-probability score for polysensitization exists in clinical practice. The qualitative alignment of the three strata with EAACI guidance on when to perform IgE testing is described in the text, with the caveat that this alignment is descriptive rather than validated.

Schäfer et al. (2025) identify probabilistic output, transparency of reasoning, and integration with existing clinical workflow as the three preconditions for clinical decision support system adoption. The Bayesian network satisfies all three preconditions at the methodological level: the output is a posterior probability rather than a binary label, the reasoning is fully traceable through the CPT chain, and the network can be queried with whatever evidence happens to be available at the point of care, degrading gracefully when some predictors are absent rather than requiring complete-case input. Collin et al. (2022) provide guidelines for computational model validation in personalized medicine and emphasise the necessity of prospective evaluation against clinical endpoints before deployment—a requirement explicitly acknowledged here and incorporated into the framework’s stated scope as a methodological demonstration rather than a validated clinical tool.

The scenarios above map the network’s inference queries onto realistic clinical decision points and are offered as concrete illustrations of operational logic, not as evidence of validated clinical utility. In Scenario C in particular—using a confirmed asthma diagnosis to update the posterior probability of IgE sensitization in a patient without IgE test results—the bidirectional inference capability provides information that no unidirectional classifier can provide without retraining a reverse-direction model. The magnitude of the update (from 0.472 to 0.651, a shift of +0.179) is clinically interpretable as moving the prior probability of IgE sensitization from 0.472 to 0.651, which remains within the intermediate stratum (0.40–0.70). A posterior of 0.651 does not cross the high-stratum boundary (>0.70); the update is therefore interpreted as a meaningful shift within the intermediate stratum rather than a stratum change, and could potentially inform consideration of further IgE testing, subject to prospective clinical validation. Whether acting on such a posterior would improve patient outcomes relative to standard-of-care clinical assessment cannot be determined from cross-sectional secondary data; prospective evaluation is required before any clinical deployment (Schäfer et al., 2025; Collin et al., 2022).

### 4.4 Uncertainty quantification and clinical communication

Hüllermeier and Waegeman (Hüllermeier and Waegeman, 2021) distinguish two types of uncertainty relevant to clinical probabilistic models. Aleatoric uncertainty is irreducible: a patient in the mid-IgE tertile with reference-range eosinophils has a posterior of 0.443 not because the model is poorly specified but because the biological process genuinely produces a mixture of sensitized and non-sensitized individuals in this marker configuration. Epistemic uncertainty reflects limited data and is visible in the bootstrap confidence intervals, which widen substantially for sparse CPT cells—the low-IgE/marked-eosinophilia cell, estimated from only 36 participants, carries considerably more parametric uncertainty than the adjacent high-frequency cells.

Kompa et al. (2021) make a broader point that applies directly here: communicating only a categorical label discards the gradient information that probabilistic models are specifically designed to produce. In this network, a posterior of 0.45 and one of 0.65 both fall within the intermediate stratum, but represent different degrees of estimated probability. Whether such differences should influence testing or other clinical decisions requires prospective validation—a distinction that a label alone cannot convey. The posterior probability, accompanied by its bootstrap interval, could serve as a potentially informative form of communication in settings where such probabilities are interpretable to the clinician.

Van Daalen et al. (van Daalen et al., 2025) show that uncertainty communication adds the most clinical value precisely where a model is least certain. In the present framework that corresponds to the intermediate stratum, where posteriors range across the full 0.40–0.70 band and bootstrap intervals are widest. A high-stratum posterior near 0.87 (high-IgE/mild-eosinophilia cell, *n* = 724) carries a narrow interval and a label whose certainty is self-evident; an intermediate-stratum posterior near 0.44 carries a wider interval and signals to the clinician that additional evidence—further testing, symptom history—could meaningfully shift the estimate.

Calibration slope = 0.9998 and intercept = −0.0001 confirm that reported probabilities accurately represent observed outcome frequencies—a prerequisite for uncertainty communication to be clinically meaningful.

These considerations suggest that the model output could be presented as a posterior probability together with its bootstrap interval, where that interval is evidence-configuration-specific rather than uniform across all patients. A stratum label alone would not capture this variation, though the appropriate form of communication in any given clinical context remains subject to prospective validation. For a patient in the high-IgE tertile with mildly elevated eosinophils (posterior ≈ 0.868), the bootstrap interval is narrow—the cell is estimated from *n* = 724 observations—and the stratum label (“high”) carries high certainty. For a patient in the low-IgE tertile with markedly elevated eosinophils (posterior ≈ 0.132), the bootstrap interval is wide because the cell is estimated from only *n* = 36 observations, and the stratum label (“low”) should be communicated alongside the caveat that this specific evidence configuration is estimated with substantially more uncertainty than the adjacent cells. This graded communication of uncertainty—itself graded by local estimation precision—is a property that a fixed binary threshold cannot provide and that *post hoc* calibration of a discriminative classifier approximates only at the population level, not at the individual evidence-configuration level (Hüllermeier and Waegeman, 2021; Kompa et al., 2021).

The distinction between aleatoric and epistemic uncertainty has direct clinical implications in this context. Aleatoric uncertainty in the mid-IgE/reference-eosinophil cell (*p* = 0.443) is irreducible—it reflects genuine biological heterogeneity among patients with intermediate IgE and normal eosinophil counts, some of whom are sensitized and some not, and no additional predictor included in the current network would fully resolve this heterogeneity without moving to allergen-specific IgE measurements. Epistemic uncertainty in the sparse cells (low-IgE/marked-eosinophilia, *n* = 36) is reducible in principle: a larger dataset would narrow the bootstrap interval for this cell, potentially changing the stratum assignment for patients in this evidence configuration. This distinction informs which cells should be prioritised for additional data collection in future validation studies (van Daalen et al., 2025; van Daalen et al., 2026).

## 5 Conclusion

This work develops and empirically evaluates a Bayesian network for probabilistic stratification of laboratory-defined serological IgE sensitization, fitted on *n* = 8,285 NHANES participants. The framework contributes four specific advances over prior Bayesian-network allergy studies: population-scale data, multi-stage immunological architecture, empirical stratum validation (13.8%/46.1%/81.6%; Figure 8), and quantified bidirectional inference (ΔAUC +0.006 with asthma evidence). The three strata correspond structurally to the three total IgE tertiles; eosinophil category provides ΔAUC = +0.010 but zero categorical reclassification. BN, LR, and RF are statistically indistinguishable in discrimination on identical predictor sets; the network’s advantage is structural. Brier score = 0.171, calibration slope = 0.9998 (95% CI 0.9563–1.0434), and intercept = −0.0001 (95% CI − 0.0522 to 0.0520) confirm excellent individual-level calibration. The model estimates serological sensitization probability—it does not model clinical allergy.

**Figure 8:**
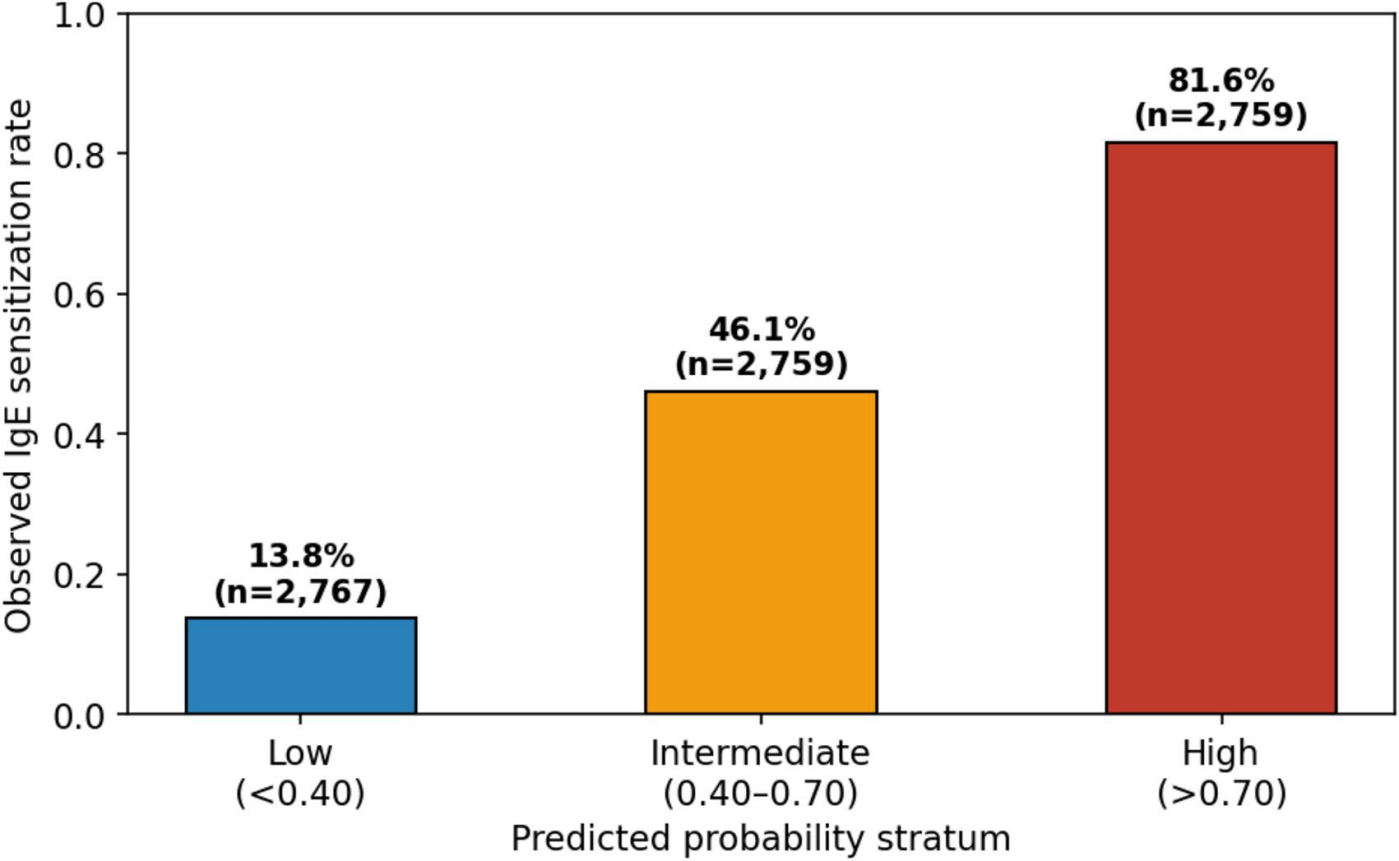
Observed laboratory-defined IgE sensitization rate by predicted probability stratum (*n* = 8,285).

Several extensions are natural next steps. External validation against an independent NHANES cycle or a cohort with longitudinal IgE and clinical outcome data would test whether the fitted CPT values are stable across populations and time periods. Empirical recalibration of the stratum boundaries via decision-curve analysis becomes possible once data distinguishing serological sensitization from clinically manifest allergic disease are available. Extending the network to incorporate allergen-specific IgE responses as individual nodes would allow modelling of the allergen-specific sensitization → exposure → symptom chain that population-level binary sensitization status cannot capture. Finally, the allergic-symptoms node currently cannot be evaluated for bidirectional inference because 72.8% of the NHANES AGQ_D questionnaire is missing; a study designed to administer the full symptom questionnaire to the immunologically characterised subsample would close this gap. These steps would progressively move the framework from a methodological demonstration toward a validated probabilistic tool for serological allergy assessment (Schäfer et al., 2025; Collin et al., 2022; Kyrimi et al., 2021; Bolt et al., 2024).

## Data Availability

The datasets analyzed in this study are publicly available and can be accessed from the National Health and Nutrition Examination Survey (NHANES) repository at the National Center for Health Statistics website (https://wwwn.cdc.gov/nchs/nhanes/). The specific data files used were from the 2005–2006 survey cycle: DEMO_D (demographics), AL_IGE_D (allergen-specific and total IgE), CBC_D (complete blood count), MCQ_D (medical conditions), and AGQ_D (allergy questionnaire). No accession numbers are required as these are publicly released NHANES data files.
